# Imaging of metabolic dysfunction in genetic cardiomyopathies

**DOI:** 10.1007/s10554-025-03470-2

**Published:** 2025-08-08

**Authors:** Giulia De Zan, Marco Guglielmo, Maarten J. Cramer, Marjolein Hol, Pim van der Harst, Birgitta Velthuis, Jeanine J. Prompers, Anneline S.J.M. te Riele

**Affiliations:** 1 Department of Cardiology, University Medical Center Utrecht, Heidelberglaan 100, Utrecht, 3584 CX The Netherlands; 2 Department of Radiology and Nuclear Medicine, University Medical Center Utrecht, Utrecht, The Netherlands; 3 Center for Image Sciences, University Medical Center Utrecht, Utrecht, The Netherlands; 4 NUTRIM Institute of Nutrition and Translational Research in Metabolism, Departments of Human Biology and Imaging, Maastricht University Medical Centre, Maastricht, The Netherlands

**Keywords:** Cardiomyopathies, Imaging, Metabolism, PET, SPECT, MRS

## Abstract

Genetic cardiomyopathies (CMPs) are a known cause of morbidity and mortality, with up to 50% of patients diagnosed below the age of 40 years for certain CMPs. With the improved availability of advanced imaging tools, significant progress has been made in early diagnosis and subsequent management. Due to the growing scientific interest in the genetic variants underlying these CMPs, data supporting a possible direct effect of the disease-defining genetic variant on cardiac metabolism have accumulated. Moreover, metabolic impairment seems to be correlated with phenotype, performance status and eventually prognosis at any stage of the disease. In this review we aim to outline the existing evidence supporting the use of imaging techniques to visualize and quantify myocardial metabolism in different CMPs. The review focuses on positron emission tomography (PET), single photon emission computed tomography (SPECT) and magnetic resonance spectroscopy (MRS), describing the basics of their functioning, strengths and weaknesses, and their use in the context of different CMPs. Finally, the latest technologies in this field and potential future directions in disease diagnosis and management are described.

## Introduction

The heart is responsible for 10% of energy consumption of the whole body [1]. While carbohydrates and fats are the main fuel, the heart has been described as a metabolic omnivore, able to degrade a vast amount of different substrates for energy production. More specifically, fatty acids (FAs), glucose, glycogen, lactate pyruvate, ketone bodies, and amino-acids, especially the branched-chain ones, can enter the Krebs cycle and contributes to adenosine triphosphate (ATP) production [2–4]. In spite of the wide range of available substrates, in normal conditions FAs are the preferred substrate by the heart for energy production, followed by glucose, but the relative contribution of FAs and glucose oxidation can change as a result of a mutual regulation known as Randle cycle [5]. In fact, one of the most important biological concepts is that the heart is able to switch energy substrate for ATP production for every given situation, such as fasting, exercise and even ischemia. This flexibility is limited in the failing heart, that can become metabolically dependent on certain categories of substrates, which can lead to increased oxidative stress and inflammation, dysregulation of cellular growth and death pathways, and, eventually to systolic and/or diastolic dysfunction [6, 7]. Meanwhile, data is growing on the potential role of specific genetic variants which may cause primary myocardial energy depletion, especially in hypertrophic cardiomyopathy (HCM) [8].

Whether metabolic dysfunction is a trigger or a result of heart failure (HF) is still a matter of debate. However, it is a definite component in the failing heart and it contributes to the worsening of cardiac function [9, 10]. Nevertheless, cardiac metabolism may still be defined as the “lost child of cardiology” [11]. For decades, myocardial metabolic imaging was primarily focused on the evaluation of ischemic heart disease, with the most notable example being the measurement of myocardial glucose metabolism by positron emission tomography (PET) to detect viable myocardium. More recently, this same imaging method broadened its indication to also detect infection and inflammation, e.g. in the setting of endocarditis and cardiac sarcoidosis. With the increase in the available data on the presence of metabolic impairment in cardiomyopathies, imaging cardiac metabolism is now regaining scientific interest. With this review, we seek to describe the available non-invasive imaging techniques that investigate the metabolic state of the heart in cardiomyopathies and explore their potential clinical applications and future role in work-up of these diseases. We will focus on genetically determined heart diseases, in which a pathophysiologic role for metabolic dysfunction is increasingly appreciated. The literature on other heart diseases including diabetic and ischemic heart diseases has already been summarized elsewhere [6, 12].

## Imaging methods

There are currently three main methods by which imaging of myocardial metabolism is possible in a non-invasive manner: PET, single photon emission computed tomography (SPECT), and magnetic resonance spectroscopy (MRS). While PET offers detailed imaging thanks to its high spatial resolution (4–5 mm), its availability is limited to larger hospitals, given the need of an on-site cyclotron or efficient logistics for radiotracer delivery. This makes SPECT the standard imaging technique in most nuclear medicine departments, despite a lower spatial resolution. Tracers developed for PET are more frequently pathway-specific, with consequent better specificity, than in SPECT. Instead, MRS does not necessarily require the use of exogenous tracers, but can also detect endogenous metabolites– such as phosphocreatine (PCr) and adenosine triphosphate (ATP). As with PET, availability of MRS is hampered by the required equipment and expertise, and its application is currently limited to research. In the next paragraphs, the principles of these techniques and their relative advantages and disadvantages are discussed.

### Positron emission tomography

**Fig. 1:**
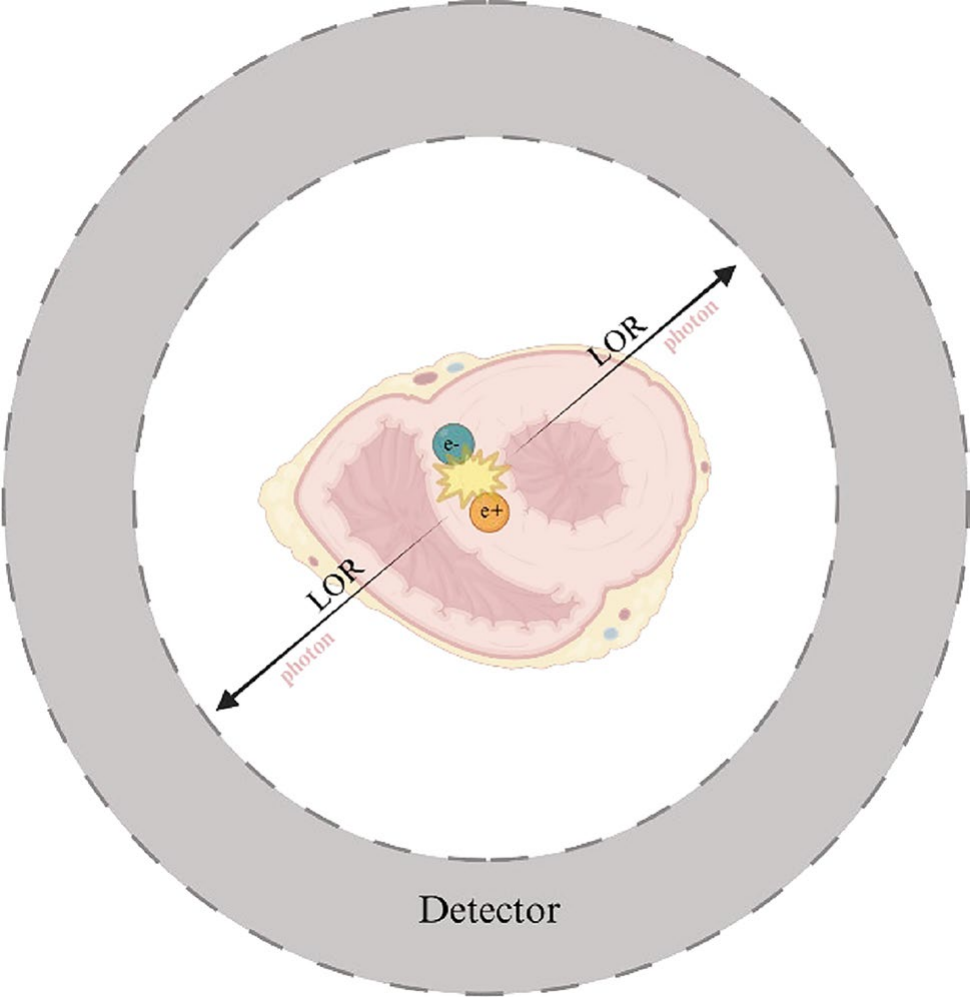
Annihilation process in positron emitting tomography. β + decay of the administered radioisotope generates one positron (e+) that annihilates with an electron (e-) resulting in two photons along opposite directions, defined as line of response (LOR). These photons are eventually detected by the surrounding detector. Created with BioRender.com.

Nuclear imaging such as PET, and SPECT, is based on the use of radiotracers. A radiotracer is a chemical compound in which one or more atoms are replaced by a radioactive isotope, such as the positron-emitting isotopes ^18^F, ^15^O, and ^11^C. Labeling an energy-providing substrate with a radioactive isotope enables to track it through its metabolic pathways. In clinical applications, radiotracers are used only in very low concentrations, yet with a high amount of energy released due to radioactive decay. Positron-emitting isotopes administered to a patient for PET imaging undergo β + decay in the body, lose energy in the surrounding tissue and annihilate with an electron. This generates two photons with 511 keV energy that are simultaneously emitted in diametrically opposite directions (Fig. 1). Two detectors facing each other detect these two photons, and the radioactivity is localized along a line between the two detectors. This is referred to as the line of response and it is determined without geometrical collimation (as required in gamma camera-based SPECT imaging) and is the reason for the excellent sensitivity of PET [13].

The most attractive feature of this technique is the ability to quantify the radiotracer kinetics, by combining knowledge of the metabolic pathways of interest with kinetic models that accurately describe the fate of the tracer in tissue. Quantitative measurements of the compound utilization are based on the specific activity of the tracer (the tracer to tracee ratio). Moreover, a wide range of both radiotracers and substrates is currently available in the clinical setting, enabling to study numerous different metabolic pathways in the heart, such as glucose uptake and utilization, by either [^11^C]-glucose or [^18^F]-fluorodeoxyglucose (FDG), or oxygen consumption, using [^11^C]-acetate or [^15^O]-O_2_ (Table 1).

**Table 1:** Quantitative measurements of myocardial metabolism that can be performed by compartmental modeling of the myocardial kinetics of various PET radiopharmaceuticals

Radioisotope	Half life	Compound	Process assessed
^15^O	2 min	O_2_	Myocardial oxygen consumption
^11^C	20 min	Acetate	Myocardial oxygen consumption
		Glucose	Glucose uptake, glycolisis, oxydation
		Palmitate	Fatty acid uptake, oxydation and storage
		Lactate	Lactate uptake and oxydation
^18^F	110 min	FDG^a^	Glucose uptake and phosphorylation
		FTHA^b^	Fatty acid uptake and oxydation

a.FDG, fluorodeoxyglucose; b. 14-(R, S)-fluoro-6-thiaheptadecanoic acid

PET also offers the potential to measure several different metabolic processes in the same imaging session by timing the administration of different radiotracers. Additionally, the short physical half-life of the radionuclides limits radiation exposure when compared with conventional single photon radionuclides, allowing for sequential studies on the same subject. Conversely, shortcomings include the need for an on-site cyclotron, or, alternatively, the difficulties in logistics for a rapid transport from the production site, the complex synthesis for many of the tracers, the high costs of PET systems and radiopharmaceutical handling, the relative complexity of image quantification requiring compartmental models, and the exposure of human subjects to ionizing radiation.

### Single-photon emission computed tomography

Similarly to PET, SPECT also uses radiotracers to image metabolic pathways. However, differently from PET, photons generated from gamma-decay are detected independently from each other. Moreover, single-photon detection uses physical collimators in order to obtain information on the direction of incident photons. Collimators have a great number of holes for the access of incident photons, but only in a direction parallel to the holes. As a result, photons reaching the collimator in other directions have a very high probability of being absorbed by the material of the collimator, partially accounting for the lower sensitivity of SPECT in comparison to PET. Because of its low sensitivity, fewer tracers have been specifically developed for SPECT when it comes to metabolic imaging. For instance, no specific SPECT radiotracers are currently available to measure myocardial glucose metabolism, for which one needs to rely on [^18^F]FDG PET. SPECT radiopharmaceuticals for the study of cardiac metabolism include instead radio-iodinated straight long-chain FAs, such as [^123^I]−15-(piodophenyl) pentadecanoic acid ([^123^I]-IPPA), and branched FAs, of which [^123^I]-b-methyl-p-iodophenyl-pentadecanoic acid ([^123^I]-BMIPP) is the most widely used (Table 2).

**Table 2:** SPECT radiotracers used for the noninvasive assessment of cardiac metabolism

Radioisotope	Half life	Compound	Pathway Assessed
^123^I	13.3 h	IPPA^a^	Fatty acid uptake, oxydation and storage
^123^I		BMIPP^b^	Fatty acid storage

a.p-123I-iodophenylpentadecanoic acid; b.123I-β-methyl-iodophenyl pentadecanoic acid

Straight long-chain FAs enter the mitochondria and are metabolized by beta-oxidation immediately, whereas [^123^I]-BMIPP is not initially metabolized via beta-oxidation because the methyl substitution precludes the formation of the ketoacyl coenzyme A intermediate. This results in prolonged retention of [¹²³I]-BMIPP within cardiomyocytes, making it well-suited for the longer acquisition times required in SPECT imaging [6]. At the same time, ^123^I is cyclotron-produced but with a longer half-life of 13 h in comparison to 110 min of ^18^F. This allows the centralized distribution of the radiotracer, as with a conventional radiopharmaceutical. Because of the lower costs and easier logistics related to the needed equipment and the radiotracers production and handling, SPECT is usually more easily available in both clinical and research settings than PET. Moreover, the assessment of more than one metabolic process is possible if a radionuclide having different primary photon energies is used for serial imaging of the heart. The major drawback of SPECT in comparison to PET is not only its worse spatial resolution (8–12 mm versus 4–5 mm), but also the inability to quantify cellular metabolic processes. This is particularly relevant, because alterations in myocardial metabolism in (non-ischemic) cardiomyopathies may not display regional disparities, making the determination of abnormal metabolism challenging. In other cases, possible regional differences in cardiac metabolism may not be detectable by SPECT due to its suboptimal resolution. Similarly, quantification of substrate delivery is not possible using SPECT, as myocardial blood flow (MBF) is typically not quantifiable. More recently, the newly developed cadmium zinc telluride detectors for SPECT enables fast dynamic acquisition, allowing the imaging of rapid radiotracer distribution in the myocardium [14]. However, while promising, its availability and implementation in clinical practice are still scarce. Additionally, the complex kinetics of the single photon radiopharmaceuticals can potentially limit the identification of the metabolic process that is being measured, such as differentiating between storage or enhanced uptake.

### Magnetic resonance spectroscopy

Cardiovascular magnetic resonance (CMR) is a widely used imaging technique in clinical cardiology, which uses the nuclear spin of protons in water (and fat) to provide detailed anatomical and functional images without the use of ionizing radiation. Magnetic resonance spectroscopy (MRS) is another application of MR, based on the same principles and making use of essentially the same hardware, however yielding different information. While CMR primarily uses tissue water for signal generation, MRS enables the detection of a wide range of metabolites due to subtle differences in the magnetic environments of nuclei in different molecules or at different sites within the same molecule, leading to different resonance frequencies. The MR signal amplitude is directly related to the metabolite’s concentration and, in combination with proper localization techniques, in vivo MRS allows for the non-invasive study of tissue metabolism. When combined with conventional CMR, measurements such as myocardial perfusion, and systolic and diastolic function are possible. Next to proton (^1^H) MRS, MRS measurements of non-proton nuclei, such as phosphorus (^31^P), carbon (^13^C) and sodium (^23^Na) yield unique information on cardiac metabolism. However, the measurement of these non-proton nuclei requires additional hardware, including nucleus-specific radiofrequency (RF) coils and a broadband specific RF transmitter (Table 3).

**Table 3:** Endogenous nuclei from which signals can be detected in magnetic resonance spectroscopy and the corresponding quantified metabolites

Nucleus	Metabolite quantified
^1^H	Total creatine, triglycerides
^31^P	ATP^a^, PCr^b^, Pi^c^, PDE^d^, pH

a. adenosine triphosphate; b. phosphocreatine; c. inorganic phosphate; d. phosphodiester

**Fig. 2:**
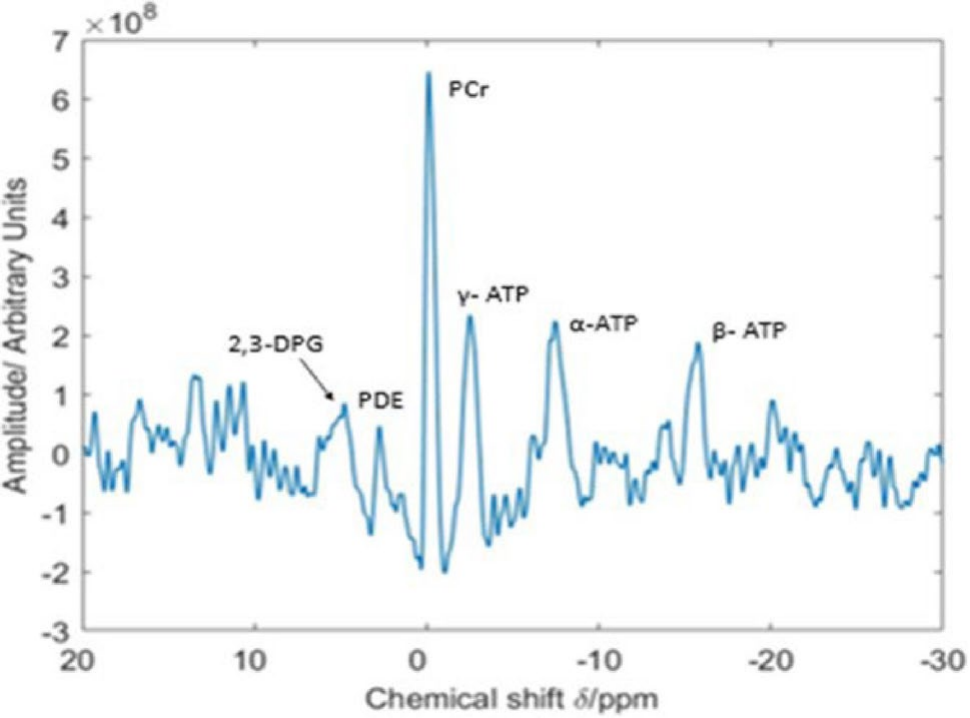
31-phosphorus magnetic resonance spectrum from a healthy volunteer. The resonances correspond from left to right to 2,3-DPG^a^ and PDE^b^, PCr^c^, and the three atoms of ATP^d^ (γ, α and β); (a) 2,3-diphosphoglycerate; (b) Phosphodiester; (c) Phosphocreatine; (d) adenosine triphosphate [117]

One of the main limitations of MRS is its low sensitivity. As a result, the long scan times and low spatial resolution of MRS severely limit its current clinical application. This is especially true for ^13^C-MRS because of the low natural abundance of ^13^C in cells, thus requiring exogenous ^13^C-labelled precursors to detect a signal. Protons have the highest MR sensitivity, but cardiac ^1^H-MRS is technically challenging due to the motion of the heart, necessitating synchronization of the ^1^H-MRS measurement with both the heartbeat and respiration. Nevertheless, ^1^H-MRS has been shown to be a valuable technique in cardiac research, allowing quantitative, non-invasive assessment of myocardial total creatine and triglyceride content in the human heart, validated by ex vivo measurements in cardiac tissue samples [15]. ^23^Na-MRS can be used to investigate and identify myocardial infarction and viability, as the total ^23^Na signal is elevated in scar, but it has no current application in imaging myocardial metabolism. While still far from the level of clinical implementation, ^31^P-MRS is at the moment the most widely used and investigated technique to study cardiac metabolism. A typical ^31^P spectrum from a healthy subject shows resonances from the three phosphate groups (alpha, beta, and gamma) of adenosine triphosphate (ATP), phosphocreatine (PCr), 2,3-diphosphoglycerate (from erythrocytes), and phosphodiesters (from membrane and serum phospholipids) (Fig. 2).

In the heart, creatine kinase (CK) acts as a temporal and spatial energy buffering system, ensuring efficient and rapid responses to fluctuating energy needs [16]. In the mitochondria, the mitochondrial CK facilitates the transfer of a phosphoryl group from ATP to PCr, and PCr subsequently transports the phosphoryl group from the mitochondria to the cytosol. There, cytosolic CK catalyzes the transfer of the phosphoryl group back to ATP, ensuring its availability for energy-intensive processes, including the activity of contractile proteins. When the demand for ATP outweighs ATP synthesis (such as in ischemia), PCr levels fall, resulting in a low PCr-to-ATP ratio. ATP levels only fall when PCr levels are substantially depleted, because the CK equilibrium constant strongly favors ATP synthesis above PCr synthesis. Cardiac ^31^P-MRS allows quantification of the cardiac PCr-to-ATP ratio, thus provides an index of the energetic state of the heart. The cardiac PCr-to-ATP ratio of healthy subjects ranges between 1.1 and 2.5, with some evidence for a decrease with increasing age [17, 18]. This large variation reflects methodologic variation of acquisition and analysis techniques, emphasizing the need for development and standardization. In healthy subjects, PCr-to-ATP ratios remain constant under exercise or, more generally, in stressed conditions, except under extreme pharmacologic stimulation, when there is a modest reduction [19]. However, the cardiac PCr-to-ATP ratio does not distinguish whether changes arise from alterations in PCr, ATP, or both. If both metabolites change in the same direction, the ratio may remain unchanged, potentially masking underlying abnormalities. Although this limitation can be mitigated by absolute quantification, steady-state metabolite concentrations still do not provide direct information about the rate of ATP synthesis. Cardiac ^31^P-MRS with saturation transfer enables quantification of the rate of ATP synthesis via CK, from which CK flux can be derived [20].

## Current evidence of metabolic dysfunction in cardiomyopathies

### Hypertrophic cardiomyopathy (HCM)

Preclinical data suggest that pathogenic variants in HCM-associated genes affect actin gliding velocity, intrinsic force, and ATPase activity, eventually leading to a higher energy demand [21]. In vitro models have provided evidence that ATP utilization for force production is higher in cardiac tissue from HCM mice and human HCM patients harboring pathogenic sarcomere variants compared to control tissue [22, 23]. Inefficient energy utilization for force generation has thus been suggested as a primary consequence of HCM-associated sarcomere variants, leading to adverse remodeling [24, 25].

**Fig. 3:**
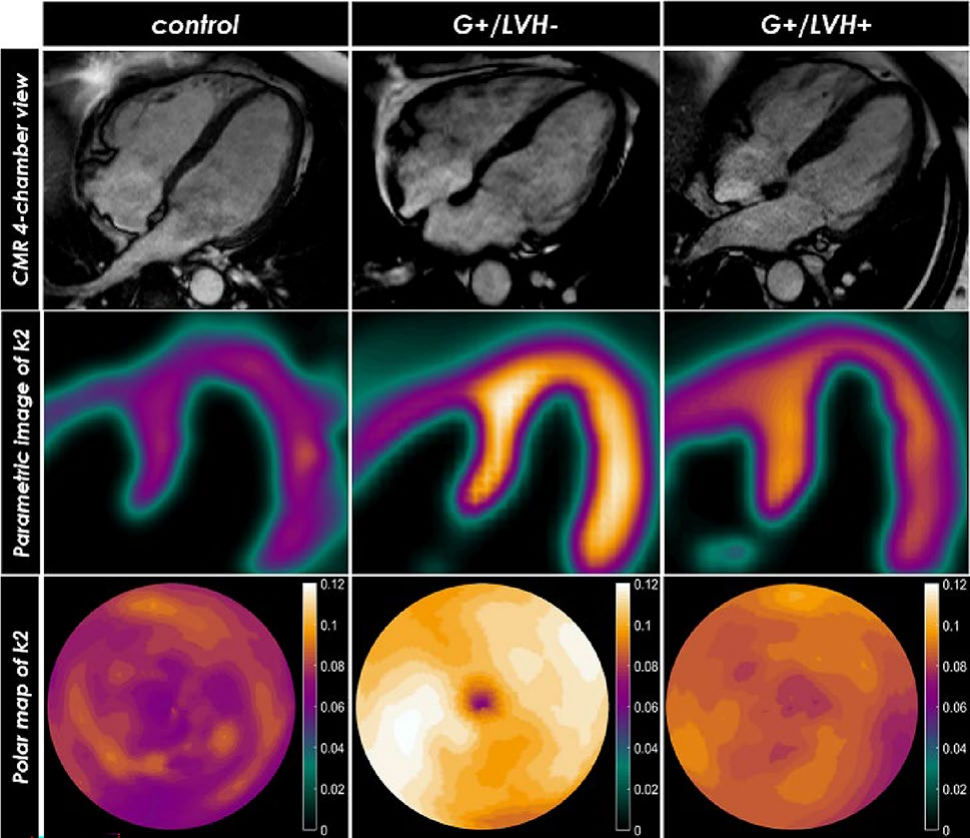
[^11^C]-acetate positron emission tomography for quantification of oxygen consumption. Imaging of a control, a genotype positive/phenotype negative patient, and a patient with HCM^a^. CMR^b^–derived 4-chamber view and [^11^C]-acetate positron emission tomographic–derived average [^11^C]-acetate clearance rate constant (k_2_) with corresponding polar maps. Myocardial oxygen metabolism was lower in patients with HCM, whereas it was increased in genotype positive/phenotype negative patients compared with controls; (a) hypertrophic cardiomyopathy; (b) cardiac magnetic resonance [118]

These findings are also reflected in imaging studies. In the last few years, [^11^C]-acetate PET imaging has been the method of choice to quantify oxygen consumption in HCM patients, which may be used in combination with conventional CMR to measure myocardial efficiency [26]. Acetate is completely metabolized in the Krebs cycle where it is oxidized and the [^11^C]-activity is converted to [^11^C]-labelled dioxide [27]. Since the heart is an aerobic organ, relying almost exclusively on oxidation of metabolic substrates for the generation of energy, acetate clearance rate, commonly known as constant k2, equals the oxidative flux through mitochondria and thus myocardial oxygen consumption (MVO_2_) [28, 29]. Subsequently, myocardial external efficiency (MEE) is calculated starting from the ratio between external work, which is the product of stroke volume and mean arterial pressure, and MVO_2_. Studies conducted using [^11^C]-acetate PET in HCM have however led to discrepant results. While early studies suggested lower oxygen consumption in affected HCM patients, more recent data indicate that early stage HCM shows an increased oxygen demand, which eventually regresses with the development of more severe hypertrophy [30–33] (Fig. 3). These discrepancies are most likely related to differences in study design. Tadamura et al. suggested a lower MVO2 in HCM patients based on their results comparing hypertrophied and non-hypertrophied segments. While they demonstrated a lower MVO2 at the [^11^C]-acetate PET in hypertrophied segments, the study did not include a healthy control group [31]. Later on, Tuunanen et al. performed a similar analysis but including healthy subjects as controls. In this case non-hypertrophied segments of HCM patients still had higher oxygen consumption than hypertrophied segments, but also higher than in the control group [32].

Noteworthy, Gurlu et al. demonstrated a lower MEE in pathogenic variant carriers, caused by higher MVO_2_ combined with lower external work. At the same time, O_2_ consumption per gram tissue was reduced in patients with a definite HCM diagnosis, who also had an even lower MEE [34]. The initial increase and subsequent reduction in MVO_2_ during disease progression could mean that the initial mutation-related increase in oxygen utilization is compensated in the hypertrophied heart by secondary mechanisms. Moreover, when compared to patients with aortic valve stenosis, no significant change in oxygen consumption and MEE was observed in HCM patients after septal myectomy, whereas all parameters significantly improved after aortic valve replacement in patients with aortic valve stenosis [34]. This further suggests that energy deficiency is a primary event that triggers secondary (mal)adaptive mechanisms leading to remodeling of the HCM heart.

The lower MVO_2_ in more advanced HCM may be explained by different reasons. First, an increased diffusion distance resulting from a relative decrease in capillary density in the hypertrophic heart may reduce oxygen uptake by the cardiomyocyte. Second, advanced hypertrophy may lead to new fibrosis and subsequent hypokinesia of hypertrophied segments (as indicated by impaired circumferential shortening) which may reduce oxygen demand [35]. Finally, lower oxygen usage might be related to loss of metabolic flexibility, leading to a compensatory switch in substrate metabolism from high oxygen-consuming lipid oxidation to low oxygen-consuming glycolysis. Indeed, initial observations were obtained in children in whom genetic defects in the enzymatic pathways critical for FA oxidation caused hypertrophy [36]. In murine models, a pathogenic variant in the gene encoding a kinase that regulates FA oxidation was associated with HCM and interventions that involve inhibition of mitochondrial FA oxidation resulted in cardiac hypertrophy [37, 38]. Finally, a recent human study linked PPARα gene polymorphisms to alterations leading to a hypertrophic phenotype [39]. In HCM patients receiving [^123^I]- BMIPP-SPECT, [^18^F]FDG PET and [^11^C]-acetate PET to study FA, glucose and oxygen metabolism respectively, BMIPP uptake was more often impaired, followed by reduction of oxidative metabolism even before alterations in FDG uptake occurred [31]. Moreover, reduced BMIPP uptake developed before perfusion abnormalities as assessed by ^201^Tl SPECT, thus excluding the role of hypoperfusion in HCM as the only explaining mechanism for reduction in FA oxidation [40–43]. However, when compared with perfusion assessed via [^13^N]-ammonia PET, the severity of [^123^I]-BMIPP defects did correlate with reduced coronary flow reserve [44]. These discrepancies might also be explained by evolving myocardial perfusion abnormalities with the progression of disease severity [43, 45]. Okizaki et al. defined early HCM as normal uptake of [^99m^Tc]Tc-tetrofosmin (used to measure MBF) and [^123^I]-BMIPP, moderately advanced HCM as normal uptake of [^99m^Tc]Tc-tetrofosmin and decreased uptake of [^123^I]-BMIPP, and severely advanced HCM as decreased uptake of both [^99m^Tc]Tc-tetrofosmin and [^123^I]-BMIPP [46]. Their data showed a progressive reduction in BMIPP uptake with disease progression, together with paradoxical triglyceride accumulation in the myocardium. In fact, Tuunanen et al. used [^18^F]-fluoro-thlaheptadecanoic acid (FTHA) PET in the hypertrophied heart to demonstrate that mild hypertrophy is characterized by increased FA uptake compared to controls [32]. This hypermetabolic state regressed with advanced hypertrophy, further confirming the heterogeneous metabolic panorama depending on the stage of the disease.

Similarly, ^31^P-MRS showed potential in identifying HCM patients. In line with the association of HCM with myocardial energy depletion secondary to inefficient force generation in cellular HCM models, human studies using ^31^P-MRS have demonstrated reduced cardiac PCr-to-ATP ratios irrespective of the degree of hypertrophy [47, 48]. Interestingly, Jung et al. found that patients with a family history of HCM had lower PCr-to-ATP ratios and higher Pi-to-PCr ratios than patients without familial HCM, despite no significant difference in severity of hypertrophy [49]. These results, together with data from preclinical models, suggest a direct role of the genetic variant in metabolic impairment, rather than a secondary effect of hypertrophy and ischemia [47, 50]. Moreover, Dass et al. demonstrated a further reduction of PCr-to-ATP ratio in HCM patients during exercise compared to matched healthy controls, which was not correlated with the severity of hypertrophy, the amount of fibrosis at CMR or myocardial blood flow, suggesting a potential explanation for exercise-related diastolic dysfunction in HCM [51]. Although the myocardial PCr-to-ATP ratio was reduced in patients with LV hypertrophy, it did not differentiate between those with and without HF. In contrast, CK flux, as measured by saturation transfer ^31^P-MRS, was not only significantly reduced in left ventricular (LV) hypertrophy, but also distinguished patients with and without HF [52, 53].

Few attempts have been pursued to study metabolism in HCM models with ^1^H-MRS. Secchi et al. studied a small group of HCM patients with ^1^H-MRS and demonstrated an increased amount of myocardial lipids compared to healthy athletes. Conversely, Nakae et al. found a lower triglyceride concentration in the myocardium of HCM patients than in healthy controls [54].

### Dilated cardiomyopathy and heart failure

Several data support the hypothesis that alterations in myocardial metabolism have a role in the pathogenesis of HF and reduced systolic function [55, 56]. Animal models of HF have shown a shift in myocardial substrate metabolism towards primarily glucose use, similar to what happens in the fetal heart, to the point where fetal isoforms of contractile and calcium regulatory proteins are re-activated and expressed [57–59]. Although this shift initially reduces oxygen demand in the failing heart, a glucose-based metabolism may later result in an energy-deficient state, ultimately decreasing the heart’s contractile performance [55]. Moreover, while the shift in myocardial substrate utilization is a compensatory response to optimize energy metabolism in HF, these metabolic changes at rest can be detrimental under stress conditions, as cardiomyopathic hearts cannot further increase glucose uptake. It has been hypothesized that reduced FA oxidation may eventually lead to their accumulation in the myocardium, resulting in lipotoxicity and, again, contractile dysfunction [60].

**Fig. 4:**
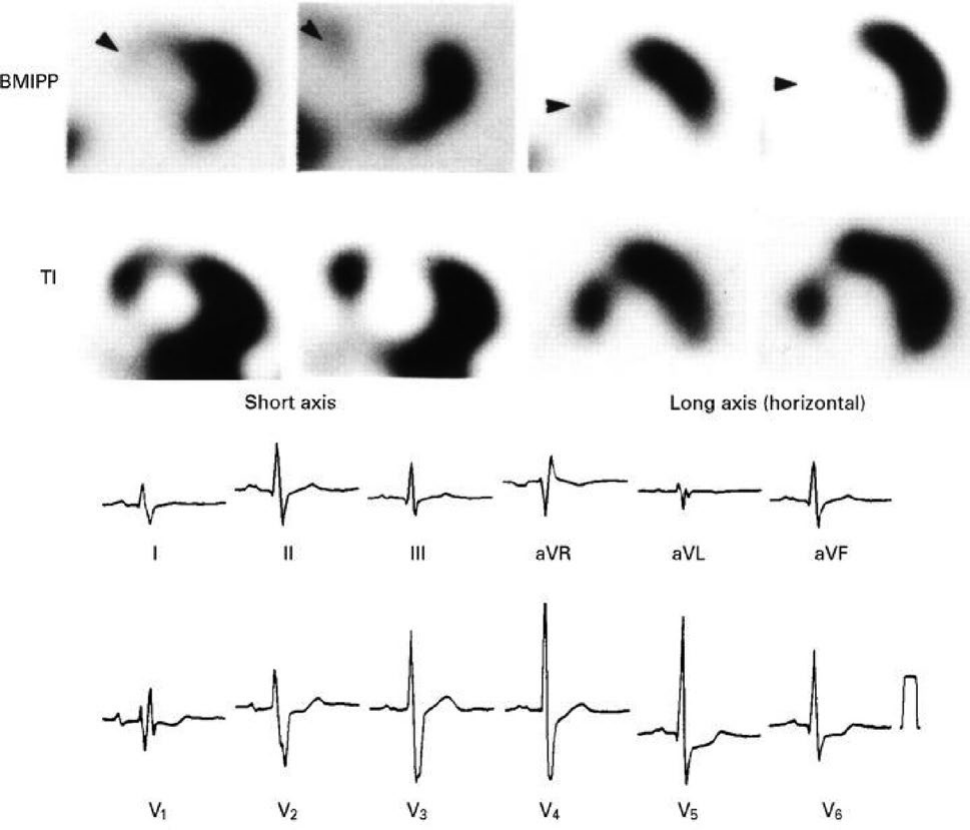
[^123^I]-BMIPP^a^ SPECT^b^ in heart failure. Short axis and horizontal images with [^123^I]-BMIPP and ^201^Tl SPECT show type B mismatching in the intraventricular septum (arrowheads) in a patient with right bundle branch block; (a) beta-methyl-iodophenyl pentadecanoic acid; (b) single photon emission computed tomography [66]

A [^11^C]-palmitate PET study in patients with idiopathic DCM showed that FA utilization, including FA oxidation, was significantly decreased, while rates of glucose utilization studied with [^11^C]-glucose PET were increased compared to normal controls [61]. This is however in contrast with the results by Taylor et al., where myocardial FA utilization, measured using [^18^F]-FTHA PET was significantly increased compared to controls [62]. While this discrepancy could be explained by inclusion of patients with ischemic cardiomyopathy, non-ischemic DCM and HF may have stage-dependent alterations in myocardial metabolism, similar to those seen in HCM. In fact, FA oxidation appears to increase in mild HF, while the switch to glucose is more typical of decompensated phases or more advanced stages [63, 64]. Interestingly, Tuunanen et al. outlined that whereas myocardial FA metabolism was indeed generally reduced compared to controls at [^11^C]-palmitate PET, further reductions in LV function were associated with insulin resistance and paradoxical up-regulation of FA uptake and oxidation [65]. Moreover, metabolic substrate switches do not seem to be related to modifications in myocardial perfusion. In their SPECT study, Yazaki et al. found that a greater decrease in [^123^I]-BMIPP than in perfusion measured as ^201^Tl uptake (defined as type B mismatching) in idiopathic DCM was associated with clinical deterioration [66] (Fig. 4).

Sasaki et al. reported that values of 0.75 or lower of cardiac uptake ratio of BMIPP to thallium could better identify patients at risk of cardiac death than LV ejection fraction in patients with non-ischemic HF [67]. Masci et al. studied DCM patients with left bundle branch block using CMR-tagging for deformation pattern, [^18^F]FDG PET for glucose utilization and [^13^N]ammonia PET for blood flow. They showed that the myocardial metabolic rate of glucose went from its lowest values in the septum (with discoordinated and poor contraction) to its highest values in lateral regions (with strong contraction), without changes in MBF [68]. Unfortunately, FA utilization was not assessed in this study. Finally, the study of Wang et al. demonstrated that increased right ventricular (RV) glucose metabolism is a common feature in patients with idiopathic DCM [69]. This study not only confirmed the relationship between RV [^18^F]FDG uptake and severity of RV dysfunction, but also demonstrated the adverse prognostic role of increased glucose metabolism in the RV as compared to the LV.

As expected, in [^11^C]-acetate PET studies, MEE was reduced in DCM, irrespective of its etiology [70, 71]. At the same time, although MVO_2_ may be similar to or even reduced in HF compared to healthy controls, it is increased in relation to MEE [72].

The reduction in PCr-to-ATP ratio in DCM measured with ^31^P-MRS is due to a reduction of the total creatin pool [48, 73–76]. Total creatin assessed by ^1^H-MRS is depleted in DCM and HCM when compared to controls, but still significantly lower in DCM than in HCM, and myocardial total creatin correlates positively with LV ejection fraction and negatively with brain natriuretic peptide (BNP) levels [77]. In fact, measurement of the absolute concentrations of high-energy phosphate metabolites has shown that in DCM both PCr and ATP are significantly reduced (51% and 35% respectively) [78]. This suggests a potential underestimation of the true impairment of myocardial metabolism in DCM if measurements are limited to the assessment of PCr-to-ATP ratio, that is also reduced, but only by 25%. Patient studies adding saturation transfer methods to ^31^P-MRS concentration measurements have demonstrated a reduction in CK flux, that was disproportionately greater than the reduction in the PCr-to-ATP ratio in patients with non-ischemic DCM [53, 79]. Bottomley et al. performed a multiple-event analysis for HF-related events including all-cause and cardiac death, hospitalization, cardiac transplantation, and ventricular-assist device placement, identifying reduced myocardial CK flux as a significant predictor of HF outcomes, even after correction for New York Heart Association (NYHA) class, LVEF, and race [52]. Despite this, the PCr-to-ATP ratio still has strong implications for risk stratification and prognosis as well. More specifically, data suggest that the PCr-to-ATP ratio has a strong correlation with NYHA class, LV ejection fraction, cardiovascular and all-cause mortality [75, 80, 81]. In addition to the indices described above, ^31^P-MRS can also be used to determine changes in the kinetics of creatine kinase shuttle, a sensitive marker of myocardial energetics. Significant reductions in myocardial creatin kinase flux were observed in patients with HF compared to healthy subjects [82].

### Cardiac amyloidosis and other cardiomyopathies

Application of imaging to study cardiac metabolism in other cardiomyopathies are scarce and mostly focus on [^18^F]FDG uptake as a surrogate for the detection and quantification of inflammation [83, 84]. Specifically, in cardiac amyloidosis nuclear imaging is mostly used for the detection of amyloid deposition within the myocardium, where cardiac scintigraphy with ^99m^Tc-labeled bone-seeking tracers, such as [^99m^Tc]Tc-PYP, has shown the highest diagnostic accuracy [85, 86]. Clemmensen et al. found that in patients with cardiac amyloidosis total myocardial oxygen consumption derived from [^11^C]-acetate PET is higher than in controls despite a reduced MEE [87]. In the same study, MVO_2_ per gram tissue correlated significantly with MBF assessed by [^11^C]-acetate PET. It is reasonable to think that the higher MVO2 in cardiac amyloidosis arises from a combination of perfusion abnormalities and structural damage due to the amyloid deposition. Moreover, subjects with light chain amyloidosis were included, who are believed to have a higher oxidative stress due to circulating light chain-mediated cellular damage [88]. Conversely, a recent study showed that MVO_2_ per gram was reduced in patients with cardiac amyloidosis [89]. However, considering that in cardiac amyloidosis hypertrophy is greatly mediated by interstitial amyloid deposit, oxidative metabolism per gram tissue may fail to reflect the metabolic state of the myocytes. Both studies outlined lower MEE in cardiac amyloidosis, and MEE below 15.7% was associated with a shorter survival [89]. In the same study, the forward stroke volume (FSV) to left ventricular mass (LVM) ratio assessed across different scanners and PET radiopharmaceuticals was superior to MEE and left atrium volume and LV ejection fraction on echocardiography to predict outcome. These findings suggest the potential of PET imaging in the clinical assessment of cardiac amyloidosis. Since FSV/LVM performed as a strong survival predictor, it might be used for patient stratification, or to monitor the disease and treatment response. Noteworthy, FSV/LVM can also be measured with more available imaging methods than PET, such as echocardiography, for which further studies are advisable. In a ^1^H-MRS study, the myocardial triglyceride-to-water ratio was decreased in cardiac amyloidosis and associated with the severity of myocardial thickening and systolic dysfunction, independent of age, body mass index and blood lipid levels [90]. A possible explanation is that amyloid deposit within the myocardial interstitium may hamper extracellular triglyceride deposition but, as already mentioned, the proteotoxicity of circulating light chains could also affect oxidative metabolism via oxidative stress. However, further research is needed to clear this mechanisms further.

## Clinical applications and future perspectives

While altered metabolism and its non-invasive detection with different imaging methods have been widely explored in cardiomyopathies, the correlation with strong clinical outcomes requires further investigation. Furthermore, metabolic imaging should also be studied in response to conventional HF or device therapy. For example, a significant increase in PCr-to-ATP ratios using ^31^P-MRS was observed in patients with DCM after beta-blocker initiation, suggesting that at least part of the beneficial effect of beta-blockers lies in an improvement in the energy state of the myocardium [91, 92]. Moreover, treatment with metoprolol was correlated with an improvement in cardiac efficiency on [^11^C]-acetate PET in patients with DCM [93]. In HCM, BMIPP SPECT showed an increase in myocardial FA metabolism after treatment with captopril [94]. Hirsch et al. reported that intravenous allopurinol increased PCr-to-ATP ratio, PCr concentration, and CK flux in HF patients [95]. In patients with non-ischaemic chronic HF, perhexiline increased myocardial PCr-to-ATP ratio by 30%, while also leading to symptomatic improvement [96].

More recently, the effects of cardiac resynchronization therapy (CRT) on myocardial metabolism have been investigated. A study using [^18^F]FDG PET revealed that heterogeneous glucose metabolism in the LV myocardium (lateral wall > septal wall) is rendered homogeneous by CRT [97]. Studies using [^11^C]-acetate PET have reported that CRT leads to increased MEE and more homogeneous MVO_2_ [98–100].

Interestingly, dietary weight loss intervention was also reported to improve myocardial energetics defined as a reduction in CK flux towards normal values in a group of patients with DCM and obesity that had an abnormally high CK flux [101].

Apart from conventional therapies, there is evidence that metabolic interventions may help to improve systolic function and MEE, especially in DCM, stimulating the interest towards novel treatments for HF [102–105]. For example, it has been proposed that the presence of insulin resistance in DCM patients further exacerbates energy depletion because of the myocardial preference for glucose as an energy substrate in HF. Recently, it has been shown that administration of glucagon-like peptide 1, an insulin sensitizer, results in increased myocardial insulin sensitivity and glucose uptake in an animal pacing model of HF [106]. These metabolic changes were accompanied by significant improvement in LV contractile function. Similarly, the administration of pyruvate to stimulate glucose oxidation improved contractility in myocardial muscle strips of DCM by increasing intracellular Ca^2+^ transients and Ca^2+^ sensitivity [107].

These data, along with the successful growth of metabolic imaging, will require advances in several areas before its widespread clinical use can be advocated. For example, accurate tracer quantification may be possible with newer SPECT systems where accurate attenuation correction can be performed. Advances in PET detector design and post-detector electronics will improve counting statistics, which should enhance the ability to perform more complex compartmental modeling, permitting more complete characterization of the metabolism of the given substrate. Moreover, hybrid systems such as PET/MRI will permit the near-simultaneous assessment of perfusion, fibrosis and metabolism, which is of key importance in various scenarios, such as viability assessment, cardiac sarcoidosis, myocarditis. However, despite its attractive features and reduced radiation dose, higher costs and low availability significantly limit the use of the PET/MRI in clinical practice, since large clinical trial are often not possible.

Regarding PET and SPECT, the key need is the development of new radiopharmaceuticals for a more comprehensive understanding of myocardial substrate metabolism. This includes radiopharmaceuticals for key regulatory sites such as for substrate transport (e.g. GLUT-1 and GLUT-4) or switching (i.e., AMPK or PPAR), for the assessment of other aspects of carbohydrate metabolism such as lactate or pyruvate, for the evaluation of the effects of altered myocardial substrate use, such as the activation of the nitric oxide system or the induction of apoptosis.

**Fig. 5:**
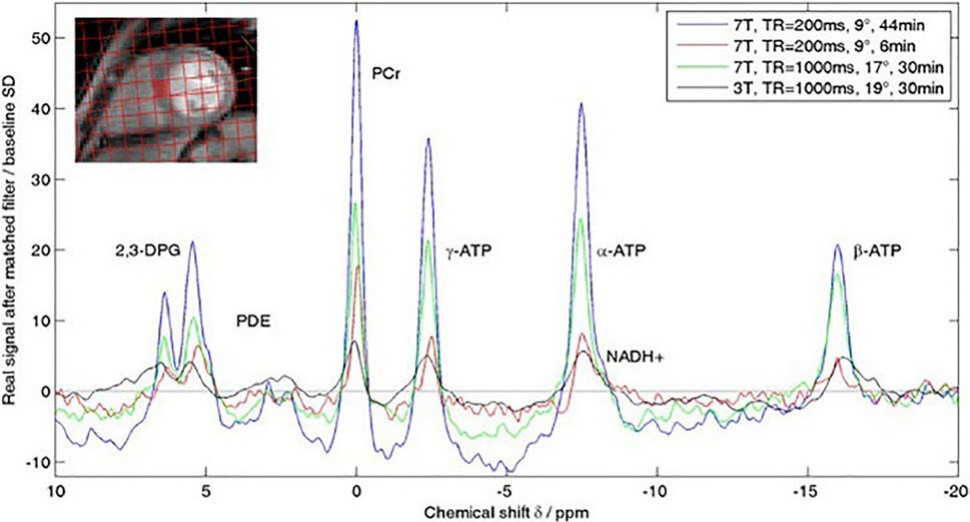
Comparison of ^31^P MR spectra at 3 Tesla and 7 Tesla. The spectra show a gain in signal-to-noise ratio of a factor of 2.8 using the same scan time or a similar signal-to-noise ratio with shorter scan time [109]

As magnetic field strength continues to increase for whole-body MR systems, the potential for MRS-based characterization of myocardial substrate use in vivo will also grow, especially for ^31^P and ^23^Na. Recent ^31^P-MRS data show a better reproducibility and signal-to-noise ratio of 7 T systems compared to 3 T, with consequently higher spatial and temporal resolution [108, 109] (Fig. 5).

A substantially higher temporal resolution will enable dynamic regional changes in metabolites to be investigated, e.g., during stress protocols. The most advanced recent developments in ^13^C-MRS are efforts to massively increase the signal by dynamic nuclear polarization (or hyperpolarization), which can boost the signal by a factor of up to 100,000 [110, 111]. Even though the spatial resolution of hyperpolarized ^13^C imaging is still relatively low in comparison to PET, PET gives no information about the metabolic fate of the substrate beyond its cellular uptake [112]. Hyperpolarized ^13^C-MRS results instead in images showing not only the uptake of metabolic substrates but also their conversion into downstream products. In this regard, the injection of [1-^13^ C]pyruvate has been used to study the pyruvate dehydrogenase complex flux, by measuring the ^13^C-bicarbonate signal [110]. Considering the growing evidence implicating a role of pyruvate dehydrogenase complex in determining contractile function, the ability to assess its flux could provide important insights into disease pathogenesis [113]. Despite its promising results, cardiac contractility, flow disturbance, the effect of the nearby lung and the need for extra hardware make cardiac application of hyperpolarized ^13^C-MRS in the clinical setting still challenging [114]. Advances in terms of improved methods for acquisition and reconstruction, hardware configuration, and design will therefore be a crucial component to delineate its future potential. More recently, deuterium MRS has been introduced to measure the metabolism of deuterated substrates [115]. Early results in healthy mice show a preference of the myocardium for acetate over glucose as substrate and demonstrate the potential of deuterium MRS to assess myocardial tricarboxylic acid cycle activity [116].

## Conclusions

Metabolic abnormalities play a recognized role in the pathophysiology of genetic cardiomyopathies and several imaging tools are available to better characterize their metabolic phenotype. However, comparative effectiveness research and technological advancement are needed to further establish the value of metabolic imaging and facilitate its translation into clinical practice for diagnosis, risk assessment and response to therapy.

## Data Availability

No datasets were generated or analysed during the current study.
